# Dentate gyrus μ-opioid receptor-mediated neurogenic processes are associated with alterations in morphine self-administration

**DOI:** 10.1038/s41598-018-37083-8

**Published:** 2019-02-06

**Authors:** Haolin Zhang, Meng Jia, Xue-Wei Wang, Can Ye, Yijing Li, Na Wang, Felice Elefant, Hui Ma, Cailian Cui

**Affiliations:** 1 Department of Neurobiology, School of Basic Medical Sciences, Key Laboratory for Neuroscience of the Ministry of Education and National Health and Family Planning Commission, Neuroscience Research Institute, Peking University, 38 Xueyuan Road, Beijing, 100191 China; 2 Department of Biology, Drexel University, 3245 Chestnut Street, Philadelphia, 19104 USA

## Abstract

Adult hippocampal dentate gyrus (DG) neural stem cells (NSCs) continuously undergo proliferation and differentiation, producing new functional neurons that remodel existing synaptic circuits. Although proliferation of these adult DG NSCs has been implicated in opiate dependence, whether NSC neuronal differentiation and subsequent dendritogenesis are also involved in such addictive behavior remains unknown. Here, we ask whether opiate exposure alters differentiation and dendritogenesis of DG NSCs and investigate the possibility that these alterations contribute to opiate addiction. We show that rat morphine self-administration (MSA), a paradigm that effectively mimics human opiate addiction, increases NSC neuronal differentiation and promotes neuronal dendrite growth in the adult DG. Further, we demonstrate that the μ-opioid receptor (MOR) is expressed on DG NSCs and that MSA leads to a two-fold elevation of endogenous MOR levels in doublecortin expressing (DCX^+^) NSC progenies in the rat DG. MOR expression is also detected in the cultured rat NSCs and morphine treatment *in vitro* increases NSC neuronal differentiation and dendritogenesis, suggesting that MOR mediates the effect of morphine on NSC neuronal differentiation and maturation. Finally, we show that conditional overexpression of MOR in DG NSCs under a doxycycline inducible system leads to facilitation of the acquisition of MSA in rats, without affecting the extinction process. We advocate that targeting MOR selectively in the DG NSC population might offer a novel therapeutic intervention for morphine addiction.

## Introduction

Accumulating evidence shows that neurogenesis comprising both proliferation and differentiation exists in the adult brain of mammals, particularly in the subgranular zone (SGZ) of the dentate gyrus (DG) and the subventricular zone (SVZ) near the lateral ventricles^1^. Many endogenous and exogenous factors can regulate adult neurogenesis and solid evidence suggests that both involuntary and voluntary opiate intake modulate neurogenesis in the hippocampal DG that in turn, alters the rewiring of neuronal circuits leading to cognitive impairment^2–7^. NSC neuronal differentiation contributes to the functional integration of neuronal precursors into existing synaptic circuits, thus modulating synaptic plasticity^8,9^. Nevertheless, previous studies attempting to elucidate the effect of opiate administration on adult neurogenesis have primarily focused on NSC proliferation rather than neuronal differentiation^2–7^. Studies on neuronal differentiation in opiate addiction that do exist are limited in that they utilize either morphine pellet implantation^2,3,5^ or morphine intraperitoneal injection^10,11^, both paradigms that do not effectively model human opiate addiction. Further, to date, these studies^3,10^ all label NSCs after opiate administration, and thus, opiate-induced effects on NSC neuronal differentiation and subsequent dendritogenesis during drug exposure currently remain unknown.

With regards to the mammalian response to morphine stimulation, opioid receptors in the brain are considered to mediate morphine-induced neuronal plasticity that contributes to the drug addiction process^12^. It is generally known that opiates (such as morphine and heroin) can mimic endogenous opioid peptides and interfere with the homeostasis of the endogenous opioid system^12^. There are 3 types of opioid receptors, μ-opioid receptor (MOR), δ-opioid receptor (DOR) and κ-opioid receptor (KOR) in the brain, among which MOR shows the highest affinity with morphine^13^ and plays major role in morphine addiction. MOR is broadly expressed in the brain^14^ and non-conditional MOR-knockout mice display decreased MSA behavior^15^. However, a potential role of the DG NSC-specific MOR in neuronal differentiation and opiate addiction remains unclear.

In the present study, we ask whether opiate exposure alters neuronal differentiation and subsequent dendritogenesis of DG NSCs via the MOR opiate receptor and explore the possibility that these alterations contribute to opiate addiction behaviors. To overcome limitations on previous paradigms used to study NSC differentiation in opiate addiction, here we use a rat morphine self-administration (MSA) that effectively mimics human opiate addiction. We show that in response to MSA, rats show an increase in NSC neuronal differentiation and dendrite growth in the adult DG, in parallel with a two-fold elevation of the NSC MOR. *In vitro* results using NSCs suggest that MOR mediates the effect of morphine on NSC neuronal differentiation and dendritogenesis. Finally, we show that conditional overexpression of MOR in DG NSCs under a doxycycline inducible system leads to facilitation of the acquisition of MSA in rats. Our findings shed light on the ongoing efforts to understand the opiate addictive processes and support the concept that selectively targeting MOR in the DG NSC population might offer a novel therapeutic intervention for morphine addiction.

## Results

### Morphine self-administration increases neuronal differentiation and dendritogenesis in the adult rat dentate gyrus

**Figure 1:**
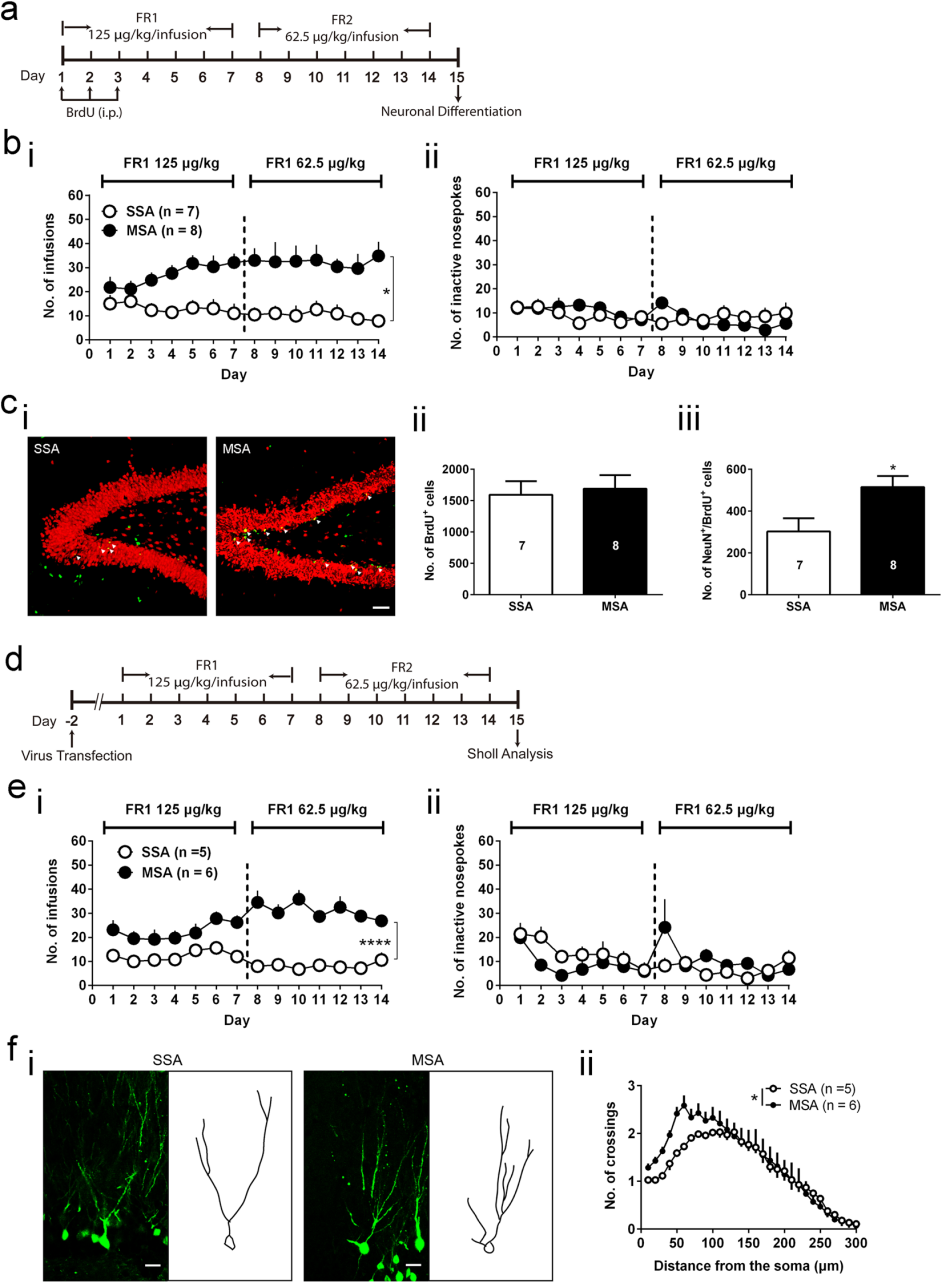
MSA increases adult DG neuronal differentiation in rats. (**a**) Schematic diagram outlining the experimental procedures of MSA and neuronal differentiation assay. (**b**) Rats acquire a stable preference for the morphine-coupled hole. (i) Infusions of SSA and MSA. Treatment × Day: ^*^p < 0.05; two-way repeated measures ANOVA. (ii) Inactive nosepokes of SSA and MSA. (**c**) MSA increases neuronal differentiation in the adult DG. (i) Representative images in each SA group. Confocal illustration shows colocalization of the neuronal marker NeuN (*red*) and the cell division marker BrdU (*green*). Scale bar represents 50 μm. (ii) MSA does not alter the number of BrdU^+^ cells in the DG. (iii) MSA increases neuronal differentiation in the DG. Number of brains scored (number of BrdU^+^ cells examined): SSA = 7 brains (279 cells); MSA = 8 brains (338 cells). ^*^p < 0.05; unpaired student’s t test. (**d**) Schematic diagram outlining the experimental procedures of MSA and dendritogenesis assay. (**e**) Rats acquire a stable preference for the morphine-coupled hole. (**i**) Infusions of SSA and MSA. Treatment × Day: ^****^p < 0.0001; two-way repeated measures ANOVA. (**ii**) Inactive nosepokes of SSA and MSA. (**f**) MSA increases dendritic complexity of neurons in DG. (**i**) Representative images and traced counterparts in each group. Scale bar represents 20 μm. (**ii**) Sholl analysis of EGFP^+^ cells: Treatment × Distance: *p < 0.05, two-way repeated measures ANOVA. All data are presented as mean ± s.e.m.

To elucidate the effects of morphine on neuronal differentiation, we first asked how voluntary morphine intake affected the differentiation of BrdU-labeled NSCs in rat DG by 2-week MSA paradigm (Fig. 1a). We found that rats developed stable preference for morphine (Fig. 1bi; Treatment × Day: F _13, 169_ = 2.066, *p* = 0.0185, two-way repeated measures ANOVA) but not saline. These rats showed comparable levels of survived BrdU^+^ cells in the DG following pulse-chase experiments. By contrast, a 1.7-fold increase in neuronal differentiation (NeuN^+^/BrdU^+^ cells) in the granule cell layer of the DG was observed after 2 weeks of MSA (Fig. 1ciii; t = 2.596, *p* = 0.0222, unpaired student’s t test). In addition, the DG neuroblasts (DCX^+^) staining showed that 10-day MSA could significantly increase the number of neuroblasts compared with saline self-administration (SSA) (Supplementary Fig. S1).

To further investigate the effects of MSA on dendritogenesis, a neuronal maturation process following neuronal differentiation, we injected a CMV-EGFP lentivirus in the DG to label few cells at the onset of MSA and reconstructed the dendritic tree 2 weeks after viral application (Fig. 1d). Using MSA training, rats developed stable preference for morphine (Fig. 1ei; Treatment × Day: F _13, 117_ = 4.226, *p* < 0.0001, two-way repeated measures ANOVA) but not saline. Using the geometric center of each cell as the center, observation and analysis of dendritic complexity of EGFP^+^ neurons in the DG revealed that MSA significantly increased dendritogenesis in the DG compared to SSA (Fig. 1f; Treatment × Distance: F _29, 261_ = 1.618, *p* = 0.0274, two-way repeated measures ANOVA).

### Morphine self-administration increases μ-opioid receptor expression in the differentiated neural stem cell progenies

**Figure 2:**
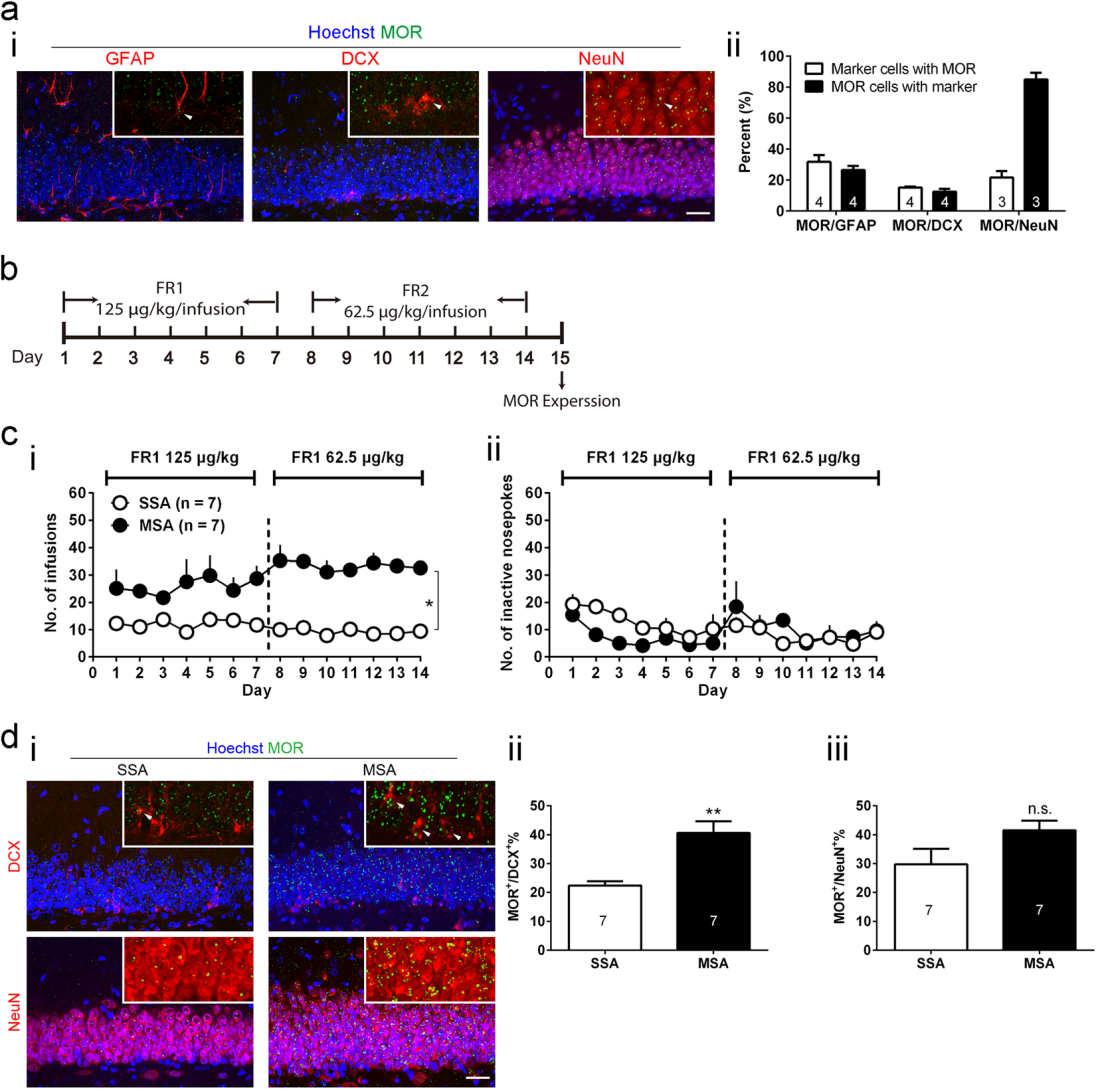
MSA increases MOR expression in the differentiated NSC progenies. (**a**) SGZ NSCs express MOR. (i) MOR screen of multiple cell markers. Scale bar represents 30 μm. (**ii**) Quantification of MOR/Marker colocalizations in the DG. (**b**) Schematic diagram outlining the experimental procedures of MSA and MOR quantification. (**c**) Rats develop a stable preference for the active (morphine-paired) hole. (i) Infusions of SSA and MSA. Treatment × Day: ^*^p < 0.05; two-way repeated measures ANOVA. (ii) Inactive nosepokes of SSA and MSA. (**d**) MSA increases the number of DCX^+^ neurons with MOR coexpression. (i) Representative images in each group. Scale bar represents 30 μm. (ii) Quantification of MOR^+^/Marker^+^ percentage: ^**^p < 0.01; unpaired student’s t test. All data are presented as mean ± s.e.m.

To explore a potential mechanism underlying morphine-induced neuronal differentiation, we first examined the expression pattern of MOR in the adult DG. The affinity of MOR antibody to its target MOR was validated by using MOR blocking peptide as negative control (Supplementary Fig. S2). Co-localization of MOR and GABAergic interneuron marker GAD67 was used as positive control (Supplementary Fig. S2). Consistent with previous studies^16–19^, GFAP^+^ NSCs located at the SGZ expressed MOR (Fig. 2a), indicating they were able to directly react to morphine stimuli. The remaining MORs within DG were either expressed on subpopulations of postmitotic neuroblasts (DCX^+^) or mature neurons (NeuN^+^) (Fig. 2ai). Quantification showed 31.76% GFAP^+^ NSCs expressed MOR, 15.16% DCX^+^ immature neurons expressed MOR and 21.67% NeuN^+^ mature neurons expressed MOR (Fig. 2aii).

Next, to validate whether increased neuronal differentiation is accompanied with elevation of MOR levels in NSC progenies, we quantified MOR expression in DCX^+^ and NeuN^+^ populations after MSA (Fig. 2b,c; Treatment × Day: F _13, 156_ = 1.906, *p* = 0.0331, two-way repeated measures ANOVA). We found that the percentage of MOR^+^ cells in the DCX^+^ NSC progenies was significantly increased (Fig. 2di,dii; t = 4.237, *p* = 0.0012, unpaired student’s t test), providing direct evidence that elevation of NSC MOR coincided with NSC neuronal differentiation. There was also a trend of percentage increase of MOR^+^/NeuN^+^ populations but not statistically significant (Fig. 2di,diii; t = 1.870, *p* = 0.0860, unpaired student’s t test).

### Morphine-induced neuronal differentiation is mediated by μ-opioid receptor

**Figure 3:**
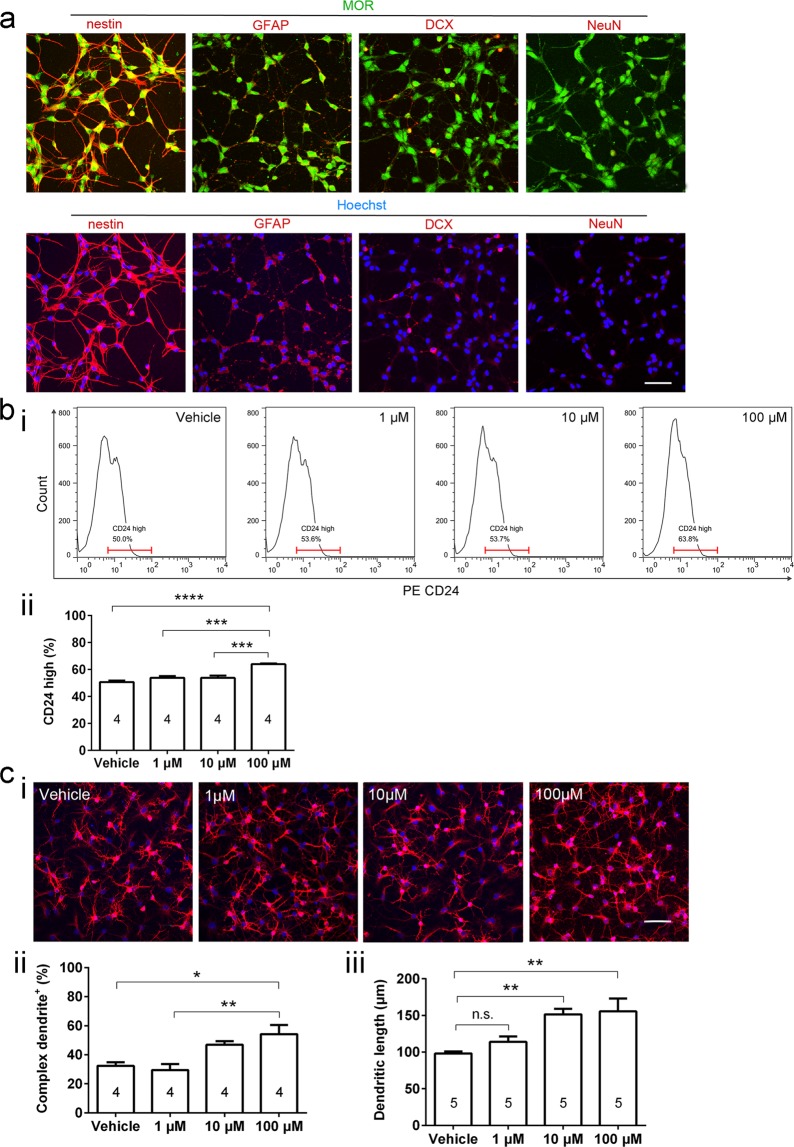
Morphine-induced neuronal differentiation is mediated by MOR. (**a**) Cultured rat NSCs express both NSC marker nestin and MOR but express negligible levels of glial marker GFAP and neuronal marker DCX/NeuN. Scale bar represents 20 μm. (**b**) FCM analysis of NSCs reveals the effect of morphine treatment on neuronal differentiation. (i) NSCs are detected by CD24. Representative FCM histograms are shown. (ii) Quantification of differentiation using fluorescence intensity. ^***^p < 0.001, ^****^p < 0.0001; one-way ANOVA. (**c**) Morphological analysis of NSCs showing effect of morphine treatment on dendritogenesis. (i) Representative images of cell morphology of NSCs after morphine treatment. Cells are labeled by neuronal differentiation marker MAP2 (*red*) and Hoechst (*blue*). Scale bar represents 20 μm. (ii) Chronic morphine treatment increases the number of cells with complex dendrites. ^*^p < 0.05, ^**^p < 0.01; one-way ANOVA with Tukey’s multiple comparisons test. (iii) Chronic and higher doses of morphine treatment increases total neurite length of NSCs. ^**^p < 0.01; one-way ANOVA with Tukey’s multiple comparisons test. All data are shown as mean ± s.e.m.

To confirm the causal relationship of MOR and neuronal differentiation, we cultured rat NSCs. Under *in vitro* conditions, there is no interference of other neurotransmitters, thus enabling us to directly assess morphine-induced effects alone. Consistent with *in vivo* MOR expression (Fig. 2a), cultured NSCs also expressed MOR (Fig. 3a). Different doses of morphine were applied to the NSCs following a chronic manner (48 hours) and neuronal differentiation was examined using flow cytometry (FCM). A well-defined cell-surface neuronal marker CD24^20^ was detected in FCM. Along with the elevated dose of morphine, neuronal differentiation was gradually increased (Fig. 3b). Statistical significance was reached with 100 μM morphine (Fig. 3b; F _3, 12_ = 23.49, *p* < 0.0001, 100 μM vs. Vehicle, q = 11.12, *p* < 0.0001, 100 μM vs. 1 μM, q = 8.473, *p* < 0.001, 100 μM vs. 10 μM, q = 8.536, *p* < 0.001, one-way ANOVA with Tukey’s multiple comparisons test), a similar morphine concentration to the theoretical circulating levels of morphine following each MSA session (129 μM), strongly supporting the biological relevance of the *in vivo* and *in vitro* systems. The circulating levels of morphine following MSA was calculated based on these average conditions: 25 infusions/rat/day, 125 μg/kg/infusion, 6.44 ml/100 g blood volume for male rats^21^ and 350 g body weight. Next, dendritic growth was examined to measure neuronal maturation. Higher concentrations of morphine (100 μM) significantly increased both the ratio of cells with secondary or more complex dendrites (Fig. 3ci,cii; F _3, 12_ = 7.920, *p* = 0.0035, 100 μM vs. Vehicle, q = 5.218, *p* < 0.05, 100 μM vs. 1 μM, q = 5.902, *p* < 0.01, one-way ANOVA with Tukey’s multiple comparisons test) and the total length of the dendrites (Fig. 3ci,ciii; F _3, 16_ = 7.563, *p* = 0.0023, 100 μM vs. Vehicle, q = 5.611, *p* < 0.01, 10 μM vs. vehicle, q = 5.193, *p* < 0.01, one-way ANOVA with Tukey’s multiple comparisons test).

### Neural stem cell specific μ-opioid receptor overexpression potentiates morphine self-administration in rats

**Figure 4:**
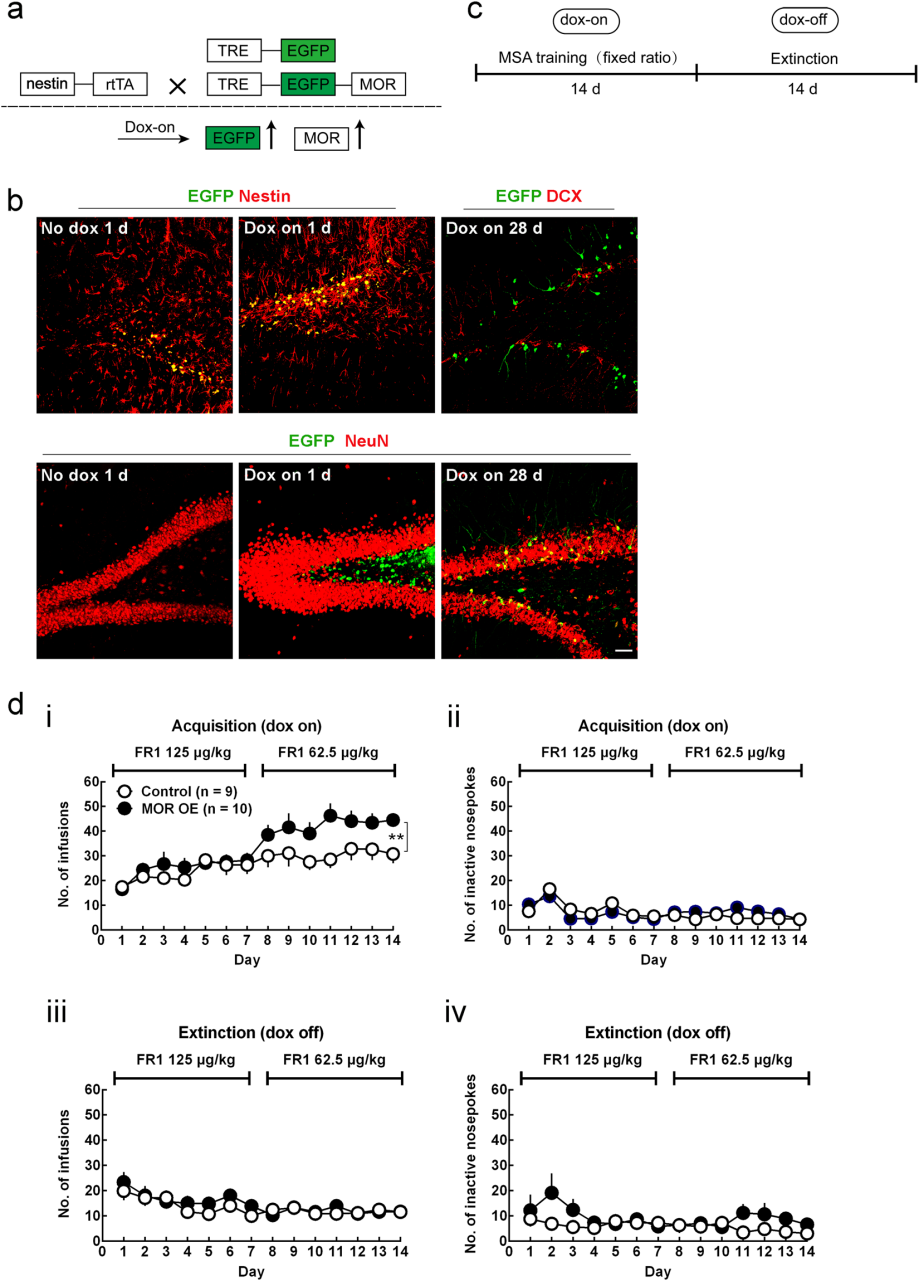
MOR overexpression in NSCs potentiates MSA (**a**) Schematic map of the lentivirus construct designed to overexpress MOR. (**b**) Dox-on for 1 day induces EGFP expression exclusively in nestin-expressing NSCs. After dox-on for 28 days, double staining reveals the cellular differentiation of the EGFP^+^ NSC lineage (upper panel: DCX, lower panel: NeuN). Scale bar represents 50 μm. (**c**) Schematic representation of experimental procedures. (**d**) MSA training. (i) NSC-specific MOR overexpression increases morphine intake during acquisition phase. Genotype × Day: ^**^p < 0.01; two-way repeated measures ANOVA. (ii) MOR overexpression rats and control rats nosepoke inactive hole at similar rates. (iii) Control and MOR dox-off rats show comparable drug seeking behavior during extinction. (iv) Control and MOR dox-off rats show comparable inactive nosepokes during extinction. All data are presented as mean ± s.e.m.

To further investigate whether there is a causal relationship between the increased DG NSC-specific MOR and opiate addiction behavior, we induced conditional MOR overexpression by lentiviral transfection. One lentivirus contained the doxycycline (dox)-regulatable reverse tetracycline-controlled transactivator (rtTA) element expressed under the control of a 1588 bp fragment of the fusion nestin gene promoter^22^, and the other one carried the MOR-1 gene controlled by the tetracycline response element (TRE) (Fig. 4a). EGFP surrogate reporter for MOR-1 expression was restricted within nestin^+^ NSCs 1 day after doxycycline induction and was unobservable in the absence of doxycycline (Fig. 4b). In addition, gene expression persisted for at least 28 days after doxycycline induction when EGFP^+^ NSCs had already developed into neurons (Fig. 4b).

MOR was next conditionally overexpressed in the DG during MSA training (Fig. 4c). The NSC-specific MOR overexpression in rats increased their morphine intake during MSA acquisition phase (Fig. 4di; Treatment × Day: F _13, 221_ = 2.382, *p* = 0.0052, two-way repeated measures ANOVA), but did not change their inactive nosepokes (Fig. 4dii). During MSA extinction, MOR overexpression was terminated due to absence of doxycycline. The MOR-overexpression rats displayed a similar decline of drug seeking behavior to the control rats (Fig. 4diii,div), indicating that morphine extinction was not affected by MOR overexpression during MSA acquisition. Taken together, NSC-specific MOR overexpression in the DG potentiated MSA acquisition in rats.

## Discussion

### The effect of chronic morphine exposure on neurogenesis

Several *in vivo*^3–6^ and *in vitro*^18,23–25^ studies documented the effect of opiates on neurogenesis or neurogenesis-like neural development in recent years, however, due to the differences in 1) drug type, 2) dose, route and/or time course of administration, and 3) animal/cell model, findings are controversial and evidence is still inadequate. Eisch *et al*. (2000) first discovered that heroin self-administration in rats, 6 hours/day for 26 days, could decrease BrdU^+^ cells in the DG^4^. Later, Kahn *et al*. (2005) reported that rats received twice daily 20 mg/kg intraperitoneal morphine injection for 1 week followed by BrdU labeling had fewer proliferating cells but more glutamic acid decarboxylase 67 (GAD67^+^) mature neurons in the DG, suggesting enhanced neuronal differentiation^6^. Contrary to the opinion that opiates can inhibit neurogenesis, increasing evidence suggests that opiates may well promote neurogenesis under chronic morphine pellet^3^ and chronic morphine i.p. injection^5,7^ paradigms, which is in line with the evidence that endogenous opioids such as β-endorphins act as neurogenesis promoters^18,26^. Since opiates mimic endogenous opioids and both generally share similar molecular mechanism, it is reasonable to speculate that opiates may also promote neurogenesis, yet may act in a different fashion involving distinct molecular mechanisms. In support of this concept, Fischer *et al*. (2008) discovered that increasing doses of chronic morphine treatment promoted NSC proliferation^5^, suggesting that different methods of morphine administration lead to different proliferation outcomes. Subsequently, in a more detailed study on NSC cell cycle, Arguello *et al*.^3^ found that using 25 mg morphine pellets for a relatively short duration of morphine exposure (24 hours) caused inhibition of cell proliferation as indicated by more G1 phase cells. Conversely, a relatively long duration of morphine exposure (96 hours) led to adaptive changes in NSCs that transformed more resting state cells from G1 to S phase thus increasing the proportion of more mature neural precursor cells (DCX^+^/nestin^+^)^3^.

Different neurogenesis detection methods may have their own limitations, as the majority of studies to date investigating neurogenesis after opiate exposure do not label the NSCs until after opiate administration^4,6,7,11,27^. Thus, under such post-drug labeling paradigms, the labeled NSCs are actually opiate-naïve and unable to represent opiate-induced effects. At the time of labeling, the NSCs previously exposed to chronic opiate might have already differentiated into neurons, and thus be too mature to be tagged by immature cell cycle markers like BrdU, therefore escaping observation. In the present study, we injected BrdU during the onset of morphine administration to capture morphine-exposed NSCs. Our results show that using a 2-week MSA paradigm promotes neuronal differentiation in the adult rat DG (Fig. 1). It should be noted here that neurogenesis is a phenomenon that includes neuronal fate determination and dendritogenesis and persists for several weeks^8^. Immature neurons may also undergo apoptosis during the highly-variable periods of the first several weeks^28^. Thus, neuron survival and dendritogenesis with prolonged follow-up of several weeks cannot be determined under our 2-week paradigm.

Our report here, along with most previous DG neurogenesis studies, consider neurogenesis in DG sub-regions as a whole. In recent years, however, the functional difference of adult-born neurons along the septotemporal axis has caught researchers’ attention^29^. The rostral/dorsal and caudal/ventral DG neurogenesis are functionally distinct and may well be relevant in MSA behavior. In this current study, we did not distinguish the relationship of rostral/dorsal and caudal/ventral DG neurogenesis and MSA, which is worth investigation in future studies.

### μ-opioid receptor-related mechanism in neuronal differentiation in the DG and morphine self-administration behavior

The existence of MOR on NSCs *in vivo* (Fig. 2a) and *in vitro* (Fig. 3a), consistent with previous reports^16–19^, implies that MOR may play a potential role in morphine-induced neurogenic processes. To validate the MOR C-terminal antibody, we performed both negative control and positive control experiments (Supplementary Fig. S2). The overall distribution of MOR in the DG shown in our current results is similar to those reported previously^14,30^. Nonetheless, the levels of MOR in GAD67^+^ GABAergic neurons are not appreciably higher than those in other MOR^+^ granule cell layer neurons. A possible explanation is that compared with the studies showing higher MOR expression in a specific interneuron subpopulation (parvalbumin^+^)^31–33^, a broader interneuron marker GAD67 covers more interneuron subtypes, with non-condensed MOR.

We found that MSA increased the NSC MOR expression in the DG (Fig. 2b–d), suggesting the participation of MOR in morphine-induced neuronal differentiation. However, our Sholl analysis of EGFP^+^ neurons following MSA includes both MOR expressing and non-expressing cells. Therefore, we cannot determine whether the increased dendritic growth is limited to the MOR expressing cells.

Causal relationship of NSC MOR and neuronal differentiation was demonstrated by morphine-treated NSCs (Fig. 3). The behavioral relevance was validated by selectively up-regulating MOR in the adult rat DG NSCs and observing the corresponding MSA behavior. We found that NSC-specific MOR overexpression during MSA acquisition significantly increased morphine intake (Fig. 4).

MOR overexpression increased both early expression of neuronal marker (Fig. 1a–c) and dendritic complexity (Fig. 1d–f), however, we were unable to determine which is responsible for the behavioral consequences of MOR overexpression. Neuronal differentiation and dendritic outgrowth, although belonging to different developmental stages, usually happen in succession. We speculate that MOR-induced neuronal differentiation and dendrite outgrowth also occurs *in vivo* and that both processes contribute to the behavioral outcomes of MOR overexpression. For future studies, it will be informative to distinguish behavioral outcomes by manipulating each process separately as well as to conditionally knock down MOR to determine whether NSC-specific MOR is not only sufficient but also required to drive MSA.

## Methods

### Animal use and care

Adult male Sprague Dawley rats (Vital River Laboratories, Beijing, China) weighing 320 g (8-week-old) were housed in a climate-controlled environment on a 12-hour reverse light/dark cycle. All of the experimental procedures were performed in accordance with the National Institutes of Health Guide for the Care and Use of Laboratory Animals, and the procedures were approved by the Animal Use Committee of Peking University Health Science Center. All steps were taken to minimize the number of rats used as well as the pain and suffering of the rats. All subjects were randomly assigned to groups.

### Morphine self-administration

Rats were implanted with a chronic indwelling intravenous catheter. 2000 units of penicillin were injected s.c. for 3 consecutive days after operation to prevent infection. For BrdU labeling experiments, adult rats were pulsed with 50 mg/kg (body weight) of BrdU (i.p.) 1 hour before and after each SA session during the first 3 days. For EGFP labeling and MOR overexpression experiments, lentivirus microinfusion was performed on the same day as intravenous catheter implantation. Rats self-administered saline or morphine (First Pharmaceutical Factory, Shenyang, China) on a standard two-hole operant paradigm in 3-hour daily sessions for 2 weeks. During MSA, the intravenous infusions of morphine were always (1) paired with the nose poking in the discriminative nose-poke hole with light stimulus inside, (2) accompanied by illumination of the cue light above the ceiling of the chamber and (3) followed by a 20-second timeout during which the discriminative light inside the nose-poke hole was off. For day 1–7 of MSA, rats self-administered morphine under the dose of 125 μg/kg/infusion^34^. For day 8–14 of MSA, the dose decreased to 62.5 μg/kg/infusion. For behavioral assay during dox-off period, rats received extinction training in the same context for another 2 weeks, whereby responses on the previously active nose-poke led to only presentation of the tone-light cue, but not morphine infusion.

### Lentivirus constructs and microinfusion

To generate inducible and conditional lentiviruses, nestin promoter^22,35,36^ was first chemically synthesized (GenScript), sub-cloned into pLV.ExSi.P/Neo-nestin-rtTA lentiviral vector and then produced by standard protocols (Cyagen Biosciences Inc., Guangzhou, China). MOR-1 (Gene ID: NM_013071) cDNA clone was purchased from OriGene (Rockville, MD, USA). MOR-1 sequence was fused with EGFP gene (720 bp) by a peptide sequence T2A and cloned into pLV.ExSi.P/Neo-TRE lentiviral vector and produced by standard protocols (Cyagen Biosciences Inc., Guangzhou, China). The lentivirus encoding TRE-EGFP was used as control.

For the MOR overexpression experiment, nestin-rtTA and TRE lentiviruses (Cyagen Biosciences Inc, Guangzhou, China) were injected to both dorsal and ventral DG. For the Sholl analysis experiment, CMV-EGFP lentivirus (GV248, GeneChem, Shanghai, China) was injected to dorsal DG. The dorsal DG coordinates were 3.6 and 6.0 mm posterior to the Bregma, 2.2 and 5.2 mm to the midline (2 × 2 arrangement), and 3.8 mm ventral from the pial surface. The ventral DG coordinates were 6.0 mm posterior to the Bregma, 5.2 mm to the midline (1 × 2 arrangement), and 6.5 mm ventral from the pial surface. Lentiviruses were dissolved in Enhanced Transfection Solution (GeneChem, Shanghai, China) with Polybrene (GeneChem, Shanghai, China) with the final titer of 1 × 10^8^ TU/ml. Infusions were made with a syringe pump connected to Hamilton syringes at a rate of 0.5 µl/min over a period of 3 minutes. The injection tip was left in place for a further 5 minutes before withdrawal. The total volume per injection site was 1.5 µl.

### Immunofluorescence

For cultured cells, the cells were fixed with 4% paraformaldehyde for 30 minutes at room temperature. Cells were then blocked and permeabilized with blocking solution (5% donkey serum and 0.3% Triton X-100) for 30 minutes at room temperature. Cells were incubated at 4 °C overnight with primary antibodies. The next day, cells were washed with PBS containing 0.3% Triton X-100 three times and incubated with the appropriate secondary antibodies for 1 hour in the dark at room temperature. The coverslips were mounted with Antifade Solution (C1210, Applygen Technologies, Beijing, China). BrdU staining required additional antigen retrieval. Heat treatment protocol was performed according to the method described previously^37^. In brief, the brain sections were incubated in 1:1 formamide/2 × SSC at 65 °C, rinsed in 2 × SSC, incubated in 2 N HCl at 37 °C and rinsed in 0.1 M boric acid (pH 8.5).

For rat brain slices, rats were anesthetized and perfused intracardiacally with 500 ml of cold saline, followed by 500 ml of 4% ice-cold paraformaldehyde in PBS. The brains were post-fixed overnight in 4% paraformaldehyde at 4 °C, then cryoprotected in 30% sucrose, and stored at 4 °C. Serial sections (50 μm of thickness) were cut through the entire hippocampus using a cryostat and stored in PBS. The remaining steps were similar to the cell staining described above.

The primary antibodies were anti-BrdU (5292, Cell Signaling Technology, Danvers, MA, USA), anti-nestin (MAB353, Millipore, Darmstadt, Germany), anti-DCX (sc-8066, Santa Cruz, Dallas, Texas, USA), anti-NeuN (MAB377 and MABN140, Millipore, Darmstadt, Germany), anti-MOR-1 (ab10275, Abcam, San Francisco, CA, USA), anti-GFAP (sc-33673, Santa Cruz, Dallas, Texas, USA) or/and anti-MAP2 (4542, Cell Signaling Technology, Danvers, MA, USA). The secondary antibodies were Cy3 donkey anti-rabbit, Cy3 donkey anti-mouse, Alexa 488 donkey anti-mouse, Alexa 488 donkey anti-rabbit, or/and Cy3 rabbit anti-goat (Jackson Immunoresearch, West Grove, PA, USA). Images were acquired using confocal laser-scanning microscope (Olympus FluoView 1000, Center Valley, PA, USA). Images were unaltered apart from brightness and contrast.

### Cell quantification in the DG

To quantify total BrdU and NeuN^+^/BrdU^+^ cells, an assumption-based approach^38^ was used to estimate the total number of cells in the DG. For each rat, from serial coronal sections (50 μm) from the entire rostrocaudal extent of the DG, every 20th section was selected for cell counting (1.8 mm, 2.8 mm, 3.8 mm, 4.8 mm, 5.8 mm, 6.8 mm posterior to the Bregma). The results were expressed as the total number of cells in the DG by multiplying the number of cells/section by the total number of 50 μm thick sections (6 sections). To quantify the percentage of MOR^+^/DCX^+^ and MOR^+^/NeuN^+^ cells, one random high-magnification (40×) field was selected from each of the aforementioned 6 interspaced brain sections. Cell countings from the 6 fields were summed for each rat and percentages of the MOR^+^/DCX^+^ and MOR^+^/NeuN^+^ cells was calculated out of the DCX^+^ and NeuN^+^ cells. All the quantifications were performed by experimenter who is blind to treatment.

### Sholl analysis

Coronal brain sections (50 μm thick) were processed for imaging. Confocal z-stacks of 3 randomly selected EGFP^+^ cells were taken from each DG section (3.6 mm and 6.0 mm posterior to the Bregma) per animal. Images of collapsed z-stacks were imported into Adobe Illustrator CS5, and dendritic trees were reconstructed using the tracing tool. Dendritic complexity was blindly analyzed from 8-bit images by using the ImageJ Sholl Analysis plug-in (http://www-biology.ucsd.edu/labs/ghosh/software/). The center of the cell’s soma was defined as the center of all concentric circles. The parameters used were starting radius (10 μm from the center), ending radius (300 μm) and interval between consecutive radii (10 μm).

### Cell culture

Rat neural stem cells (RASNF-01001, Cyagen, Guangzhou, China) were cultured following Walker T.L. and Kempermann G.’s protocol^39^. In brief, the culture medium was made by mixing Neural Basal Medium with 2% B27, 1 × GlutaMAX, 2 μg/ml heparin, 50 units/ml penicillin/streptomycin, 20 ng/ml EGF and 20 ng/ml FGF-2. The plates were coated with PDL/Laminin. The cells were grown in the form of adherent monolayer cultures and were passaged when they reached 80% confluency. For differentiation, the concentrations of EGF and FGF-2 were gradually reduced^39^. When EGF and FGF-2 both reached zero concentrations, vehicle or morphine (1 μM, 10 μM, or 100 μM) was added for 48 hours and neuronal differentiation was examined.

### Flow cytometry

Single-cell suspensions were obtained by repetitive pipetting. 30, 000 cells were used for each biological repeat. Individual biological repeats were from different plates. The qualified cells for fluorescent analyses were filtered by forward-scattered light (FSC) and side-scattered light (SSC). For cell differentiation, anti-CD24 PE (553262, BD Biosciences, San Jose, CA, USA) was used. The parameter calculated was percent of cells with higher CD24 fluorescent intensity. The gating criteria was to gate the CD24^high^ cells (50% of total) in the vehicle treated group and apply the same gate to the morphine treated group. All procedures were performed according to the manufacturer’s instructions. Data were analyzed with FlowJo 7.6.5 software (FLOWJO, Ashland, OR, USA).

### Cell morphological analyses

To analyze the proportion of dendrite^+^ cells under morphine treatment, random fields under high magnification (40× objective) were taken for cell counting. Each microscopy field was taken from an individual petri dish. Percentage of the cells with secondary or more complex dendrites was quantified.

To analyze the total dendritic length, 4 randomly selected cells from each of the random fields were measured and averaged. Length parameters of the microscopy fields were then averaged. The dendritic length was measured using Image J software.

### Statistical analyses

Shapiro-Wilk test of normality by SPSS software was made before parametric analysis. Student’s t tests were used for comparison between parameters from two groups. One-way ANOVA tests with Tukey’s multiple comparison test were used for comparison between parameters from multiple groups. Two-way repeated measures ANOVA tests were used for MSA behavior analysis and Sholl analysis.

## Supplementary information

Supplementary information

## Data Availability

The datasets generated during and/or analysed during the current study are available from the corresponding author on reasonable request.
