# Supplementary material for: Dentate gyrus μ-opioid receptor-mediated neurogenic processes are associated with alterations in morphine self-administration
Source: Sci Rep. 2019 Feb 6;9:1471. doi: 10.1038/s41598-018-37083-8 (PMC6365505; doi:10.1038/s41598-018-37083-8)

Dentate gyrus μ-opioid receptor-mediated neurogenic processes are associated with alterations in morphine self-administration

Haolin Zhang[1], Meng Jia[1#], Xue-Wei Wang[1#], Can Ye[1#], Yijing Li[1], Na Wang[1], Felice Elefant[2], Hui Ma[1], Cailian Cui[1*]

[1]Department of Neurobiology, School of Basic Medical Sciences, Key Laboratory for Neuroscience of the Ministry of Education and National Health and Family Planning Commission, Neuroscience Research Institute, Peking University, 38 Xueyuan Road, Beijing 100191, China

[2]Department of Biology, Drexel University, 3245 Chestnut Street, Philadelphia 19104, USA.

[#]Authors contributed equally to this work as second authors

[*]Correspondence: Cailian Cui, M.D. Professor

Neuroscience Research Institute

Peking University Health Science Center

38 Xueyuan Road

Beijing 100191, China

Tel: +86-10-8280 1120

Fax: +86-108280 1120

Email address: clcui@bjmu.edu.cn

Supplementary materials

Supplementary methods

Morphine self-administration

Morphine self-administration was performed for 10 days with FR1 at the dose of 125 μg/kg/infusion in 2.5-hour daily sessions. The remaining procedures are the same as the procedures in the main text.

μ-opioid receptor controls

For MOR negative control, MOR antibody was pre-incubated with an MOR control peptide (AG375, Millipore, Darmstadt, Germany) for one hour at room temperature prior to incubation with the brain sections. 10^{-5} M peptide concentration was used to block antibody binding. For MOR positive control, co-staining of MOR with a GABAergic interneuron marker GAD67 (MAB5406, Millipore, Darmstadt, Germany) was performed.

Cell quantification in the DG

The total DCX^+ cells were quantified following the same assumption-based approach as described in the main text.

Supplementary figure legends

Supplementary Figure S1. MSA increases the number of DCX^+ newborn neurons (neuroblasts) in the adult DG. (**a**) Schematic diagram outlining the experimental procedures. (**b**) Rats develope a stable preference for the active (morphine-paired) hole. (**i**) Infusions of SSA and MSA. Treatment × Day: $^*p < 0.05$; two-way repeated measures ANOVA. (**ii**) Inactive nosepokes of SSA and MSA. (**c**) MSA increases the number of

neuroblasts (**i**) Representative images in each group, indicated by the neuroblast marker DCX (*red*). Scale bar represents 20 μm. (**ii**) Quantification of DCX^+ population: total number of DCX^+ neurons. $^{**}p < 0.01$; unpaired student's t test. All data are presented as mean ± s.e.m.

Supplementary Figure S2. A negative control MOR blocking peptide and a positive control GABAergic interneuron antibody anti-GAD67 have been introduced to show MOR antibody specificity. In the DG, with blocking peptide, no MOR signal is detected; with GAD67 co-staining, MOR shows co-localization. Scale bar represents 30 μm.

Supplementary figures

Supplementary Figure S1

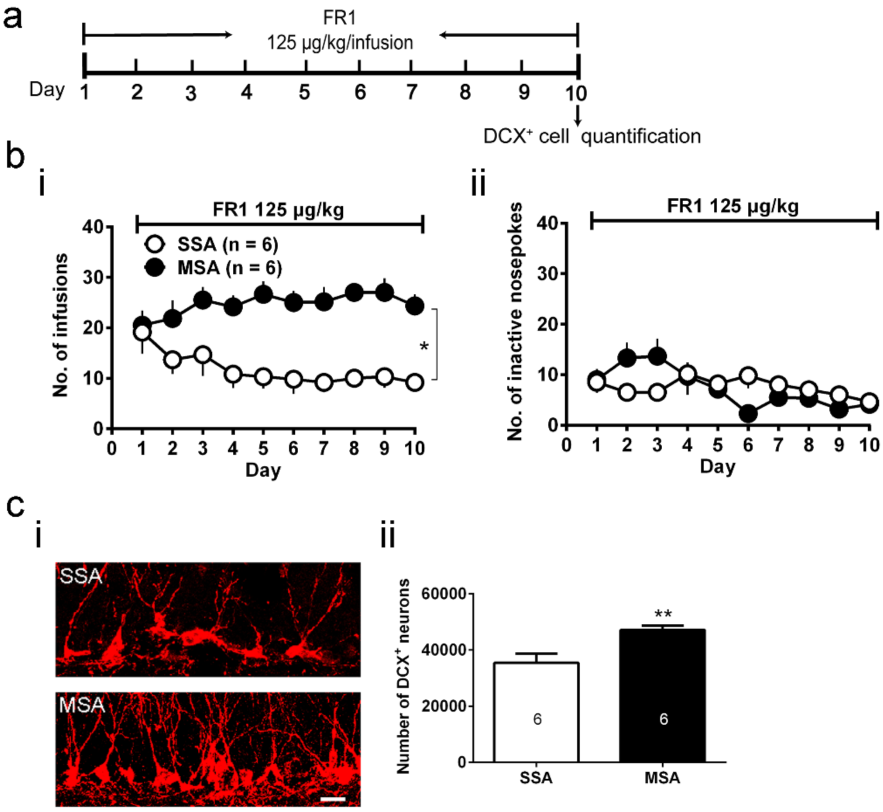

Supplementary Figure S2

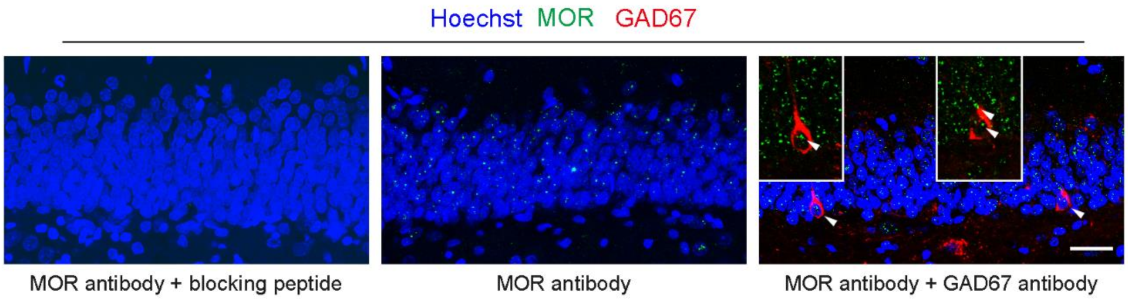

Supplement: Supplementary file 1 — Supplementary information [file 41598_2018_37083_MOESM1_ESM.pdf]
